# Leveraging Spruce Bark Particle Morphology for Enhanced Internal Bonding in Particleboard Production

**DOI:** 10.3390/polym16212988

**Published:** 2024-10-25

**Authors:** Jakob Gößwald, Marius Cătălin Barbu, Eugenia Mariana Tudor, Pavel Král

**Affiliations:** 1 Faculty for Forestry and Wood Technology, Mendel University in Brno, Zemedelska 1, 61300 Brno, Czech Republic; goess-forest@outlook.de (J.G.); pavel.kral@mendelu.cz (P.K.); 2 Design and Green Engineering Department, Salzburg University of Applied Sciences, Markt 136a, 5431 Kuchl, Austria; cmbarbu@unitbv.ro; 3 Faculty for Furniture Design and Wood Engineering, Transilvania University of Brasov, B-dul. Eroilor nr. 29, 500036 Brasov, Romania

**Keywords:** outer bark, bark fibers, spruce, larch, particleboard

## Abstract

The continuous rise in global demand for wood products has led to an increase in prices and a surge in research into alternative resources. As a byproduct of the timber industry, bark has emerged as a promising supplement in particleboard (PB) production. However, its anatomical structure, the presence of extractives, and its inferior mechanical properties complicate the production process, which have not yet been fully overcome at a commercial scale. This study proposes a paradigm shift, advocating for separate and specialized bark constituent processing in a wet state. Three bark-based raw materials—namely, outer bark particles, bark fiber clumps, and bark fibers—were investigated under varying wood content scenarios. PBs with a target density of 0.7 g/cm^3^ and a thickness of 16 mm were produced using mixtures of these bark-based materials and wood particles in different ratios bonded with a urea–formaldehyde adhesive. The results demonstrated that these bark constituents exhibit distinct properties that can be optimized through tailored processing techniques. Compared to bark fibers, outer bark particles displayed about 40% lower water absorption and thickness swelling. However, bark fibers improved the internal bond by about 50% due to their favorable morphology compared to outer bark. These findings highlight the potential of bark as a valuable resource for particleboard production and pave the way for its efficient utilization through specialized processing strategies.

## 1. Introduction

The incorporation of bark into wood-based panel production has been explored since the 1960s, with Charles Henry Burrows, who opened new ways by studying particleboards from Douglas fir bark, as one of the pioneers in this field [1]. Blanchet et al. [1] and Yemele et al. [2] delved into the utilization of bark particles for structural boards, recognizing that these composites exhibit a reduced modulus of rupture (MoR), internal bonding (IB), and modulus of elasticity (MoE), in part due to the lower cellulose content of bark in relation to wood [3]. On the flip side they offer improved thickness swelling (TS) [1,2] and reduced formaldehyde emissions [4,5]. To mitigate the inherent drawbacks of using bark, the researchers adopted various strategies, including pretreatment with hot water [2], increased glue amount, and extended pressing times [1,6] to enhance the boards’ structural integrity and usability. The use of alternative resin types like PF (phenol formaldehyde), lignin-PF and polymeric methylene diphenyl disocyanate (pMDI) in a 1:1 mixture of wood and bark was examined. pMDI, while not commonly used for particleboard manufacturing, was shown to have the best influence on mechanical and physical properties [7]. These adjustments were partly due to the higher thermal diffusivity of bark [4,8].

In response to the growing demand for thermal insulation, numerous researchers have studied the use of lower-density (0.25–0.5 g/cm^3^) bark-based insulation, including *Robinia pseudoacacia* bark [9], larch bark with bio-based resins like tanin hexamin [10,11] and spruce bark [8]. These panels are primarily designed for thermal insulation, leveraging the inherent properties of bark to offer effective insulation while also reducing environmental impact. The potential for acoustic insulation applications was also explored [12].

To achieve better fire resistance, clay boards were developed by incorporating mineral materials into the bark particle mix. This innovative approach utilizes the fire-retardant properties of both clay and bark, enhancing the safety and applicability of bark-based boards in construction settings where fire resistance is crucial [13].

Considering that particle-based wood panels tend to exhibit lower strength than fiber-based boards, ref. [14] explored the integration of wood fibers and inner birch bark particles in a three-layered structure, achieving industry-competitive strength values. This method allows the substitution of up to 78% of wood fibers on the surface and 95% in the core with more economical bark particles. Xing et al. [15,16] advanced this concept in two studies by defibrating bark for its use in the core layer, successfully replacing wood fibers. Despite these innovations, a lower IB and MoE were still observed.

Gao et al. [17] examined self-bonded panels utilizing bark fibers, partially mixed with wood fibers, where dry fiber mats were pressed at high temperatures above 200 °C and with a high compression ratio. This process harnessed self-bonding effects such as particle plastification and extractive polymerization, resulting in boards with a high density of 1 g/cm^3^ that met industry standards.

Low-density insulation boards using a wet process were prepared by Gößwald et al. [18], achieving a thermal conductivity of 0.04 W/(m·K) at a density of 0.16 g/cm^3^.

Unlike wood, which provides structural strength to the tree, the mechanical strength of bark is of lesser importance, as the load-bearing function is primarily carried out by the wood structure [19]. This corresponds to a lower cellulose content, which correlates with lower stiffness of bark-based boards [3]. However, certain physical properties, such as thickness swelling, tend to be reduced in bark-based materials due to factors like higher lignin content [3,8,15,17] and unique bark morphology. Bark is a highly specialized tissue on the living tree, serving multiple purposes including assimilate transport, protection against water loss, and defense against pests [8,20]. Bark is composed of multiple layers with distinct characteristics, making its overall structure very heterogeneous [8,17].

Bark comprises two main layers: the first is the phloem, or inner bark, which is responsible for the transport of assimilates [19,20]. The inner bark, strongly depending on the species, consists of long cells that have evolved to have increased mechanical strength to prevent cracking and ensure functionality, while still being flexible [19,20]. Due to this strength, inner bark fibers were utilized by early humans [21,22].

The outer bark, or rhytidome, does not inherently contain fibrous components but includes remnants from older phloem layers from which it originates [19]. The rhytidome often consists of multiple layers, with its cells being more polygonally shaped and containing higher amounts of suberin. Suberin is known for its hydrophobic properties, providing the bark with enhanced resistance to moisture [22]. Other functions of the outer bark include protection from pests and, in some cases, fire resistance. This is facilitated by the increased thickness of the rhytidome and the presence of protective extractives [19].

Long fibers with a high aspect ratio are known to be favorable for mechanical strength [3,23]. To maximize this potential, the processing of the fibers should be meticulously designed to effectively separate the fibers from the tissues, aiming to produce fine fibers for optimal mechanical properties [24].

On the other hand, the outer bark does not exhibit a distinct fibrous structure similar to the inner bark or the wood, making the formation of elongated particles challenging. The processing of outer bark is more likely to result in particles that are spherical or flat in shape, due to the layered nature of the rhytidome [19], which is not ideal for particle board production. However, considering the protective function of the outer bark, materials derived from it are expected to exhibit better performance in terms of thickness swelling due to its composition, which includes hydrophobic substances like suberin and lignin [3,20].

Extractives are also known to interfere with the bonding in particle board manufacturing, since, among other factors, some extractives can form bubbles when subjected to the high temperatures that typically occur during the pressing step [3].

Given the distinct compositions and properties of the inner and outer bark, separating these materials during processing could enhance the overall economic value. The respective bark components should therefore be used in applications that best suit their inherent properties. This hypothesis warrants investigation in this study to determine the feasibility and effectiveness of separate processing pathways for inner and outer bark in enhancing the performance and application potential of bark-based particleboards.

## 2. Materials and Methods

The primary raw material for this study was procured from a medium-sized sawmill located in Mühldorf, Germany, where the debarking process was conducted with a rotary drum debarker. Primarily composed of spruce bark (*Picea abies* (L.) H. Karst.) and larch (*Larix decidua* Mill.), the material may have contained minor inclusions of other bark species or wood residues, as this is common for industrial conditions. To prevent moisture loss and maintain a high moisture content, the bark was collected not only from the surface of the pile but also from its more central layers and stored in an intermediate bulk container (IBC) submerged in tap water at about 15 °C.

Subsequent processing of the bark was conducted in its wet state. This method was chosen not only to produce a fibrous material, but also to facilitate the separation of inner bark from outer bark to a certain degree. It is also noteworthy that extractives were removed in this step. The fibers were then gathered and subjected to a turbulent airstream generated by a vacuum cleaner (Kärcher NT 30/1, Winnenden, Germany) to individualize the clumped fibers.

To achieve a fraction of mostly single fibers, the wet fiber material was sieved (mesh size 4 mm) and then dried and conditioned to the equilibrium moisture content of 14% by exposure to normal climate (23 °C, 65% relative humidity). The bark processing in these preparatory stages was decisive in determining the subsequent distinctive bark morphologies from the same raw material.

**Figure 1:**
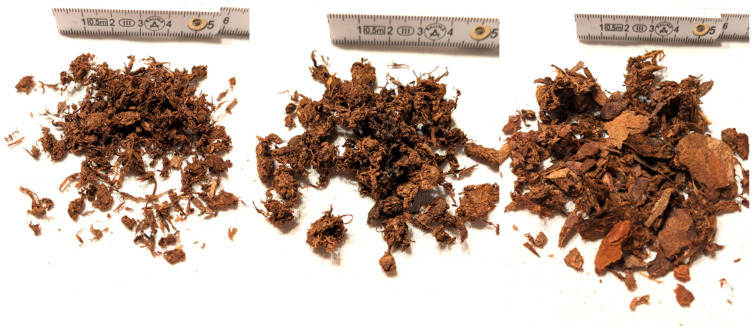
Raw material: materials F (bark fibers, **left**), FK (bark fiber clumps, **middle**), and B (outer bark, **right**).

The three main bark types under examination (bark fibers (F), fiber clumps (FK) and outer bark (B)) exhibit distinct differences in their size, shape, and composition, despite all being derived from the same source of raw bark in roughly equal proportions (Figure 1).

**Material F**: This fraction is the most refined, comprising individual fiber bundles, small-sized clumps of fibers, and reduced remnants from the outer bark. The individual fibers are characterized by their finer edges, which may enhance the material’s ability to form stronger bonds with adjacent particles and fibers due to increased surface area. The fiber clumps, which take on a more spherical shape as a result of the drying process, offer decreased surface area on one hand. On the other hand, they leverage the inherent self-bonding properties of wet fibers, potentially minimizing the amount of adhesive needed to establish a stable bond. The fibers and small fiber clumps are typically under 8 mm in size.

**Material FK**: This material predominantly consists of fiber clumps, similar in formation mechanism to those in Material F, with lengths of approximately 10 mm. In addition to these clumps, sporadic individual fibers were also generated, which may additionally be found adhering to the clumps. Leftovers from the outer bark are seldom found isolated but are more commonly encapsulated within the clumps.

**Material B**: This material is primarily composed of outer bark. The pieces of bark vary significantly in size, achieving up to 30 mm in diameter while being mostly round and flat in shape. It also includes longer, coarser bark fibers.

In addition to bark, wood particles (spruce) were also used for the core layer, with an approximate average length of 3.6 mm [25] and a comparatively high degree of slenderness. This material was obtained from the company Kaindl (Wals, Austria) and contained mostly recycled material.

For the production of bark-based boards, urea–formaldehyde (UF) resin was selected due to its widespread application in this field. Solid content: 67.1%

The resin was supplied by Kaindl company, located in Wals, Austria. Owing to the extensive sample size required for this study, board production was organized into two batches. These batches differed in the glue used: the first utilized glue nearing its shelf life (⅔ of the boards across all groups), while the second employed freshly sourced glue (⅓ of the boards across all groups). Across all samples, an 8% UF resination factor was used, with a solid content of 65%. To reduce the viscosity of the mixture to 460 MPa·s (MPas), 21% distilled water was added to the resin. The pH value was 8.6 and the condensation time was 55 s.

The mixing process involved the use of a WAM WBH 75 laboratory plug-share mixer (WAM: Ponte Motta, Italy). During this process, glue was sprayed into the mixer to ensure uniform distribution, and the mixture was blended for 5 min at 300 rpm. Towards the end of the mixing phase, 2.5% of an ammonium nitrate-based hardener was introduced. The mixture was then manually pre-compressed in a 32 cm × 32 cm wooden mold and subsequently pressed using a Höfer HLOP 280 laboratory hydraulic press (Höfer: Taiskirchen, Austria). This pressing occurred for 350 s at a temperature of 200 °C, incorporating occasional ventilation steps, to achieve a final thickness of 16 mm and a density of 0.7 g/cm^3^.

Once cooled, the boards were sanded and trimmed to the specific dimensions required for mechanical testing. Tests for modulus of elasticity (MoE) and modulus of rupture (MoR) were conducted following EN 310 [26] with four replications per board, while internal bond strength was evaluated as per EN 319 [27], with three replications per board. Additionally, water absorption and thickness swelling tests were carried out following EN 317 [28], with four replications per board. After conditioning the samples in a standard climate (20 °C, 65% relative air humidity), mechanical assessments were performed using a Zwick/Roell Z 250 universal testing machine (Ulm, Germany).

The formaldehyde content was determined using the perforator method. The samples were closely sealed after production in plastic foil and tested according to EN 120/EN ISO 12460-5 [29,30].

### Design of Experiment (DOE)

Given that a tree’s composition includes approximately 10–15% bark [1], bark will likely not replace wood completely, but rather to some level depending on its availability. Therefore, the DOE (Table 1) encompassed four levels of bark content, distributed evenly from 0 to 100%. It is important to note that at 0% bark content, the experimental design effectively simplifies to a board composed solely of wood. Consequently, we designated the F0 group to represent both FK0 and B0 scenarios.

Two additional groups were added in order to enhance comparability with existing research, referencing [8,15]. These groups are defined as follows:

P100: This group consists of larch bark sized between 2 and 4 mm, with three replications for each board.

HF50: Comprises 50% material F and 50% wood fibres, also with three replications for each board.

**Figure 2:**
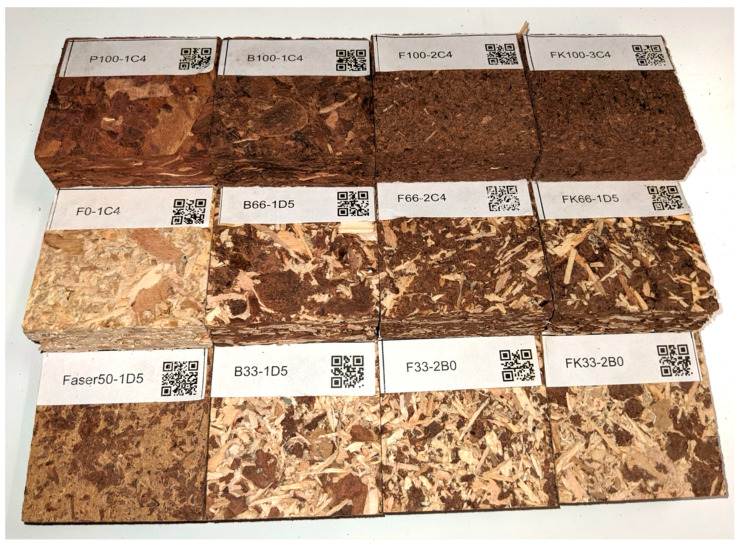
Sample images from all tested materials according to DoE.

In total, 36 boards were produced, excluding an additional four smaller panels from each group, as sources for 25 mm × 25 mm samples (Figure 2) for formaldehyde content analysis according to EN 120 [30].

Statistical analysis and graph plotting were performed using R statistical software (Version 4.2.2). After descriptive analysis, the data were corrected with robust linear regression for known covariates, like density. If assumption violations were detected, measures were taken to account for them. The residuals were then used to determine the significance between groups utilizing Tukey’s HSD.

## 3. Results

This section includes the results for the physical and mechanical properties, assessment of the impact of material type on particleboard properties and the formaldehyde content of all manufactured bark-based composites.

### 3.1. Physical and Mechanical Properties

**Figure 3:**
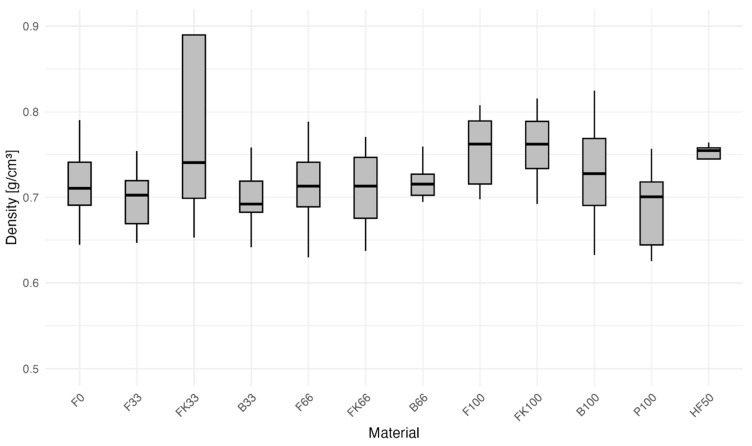
Box plot of density by group of bark-based particleboards.

Density in wood-based panels is one of the most significant and relevant variables to consider when studying panel properties. Although in this DoE the density was not explicitly varied, some variations were observed in a range of ±0.12 g/cm^3^ around the average density of 0.74 g/cm^3^. As shown in Figure 3, group FK33 was an outlier with a notably higher density and standardization of 0.89 g/cm^3^ ± 33%. This group essentially doubles the standard deviation, thus making it imperative to correct the density in the upcoming deeper analysis. In addition, a slight correlation between bark content and density, favoring bark in this aspect, can be observed. This might be due to the different moisture balance of bark and wood. Apart from a few outliers, the median is centered inside the boxes, hinting at normality and a sufficient number of samples.

The outer bark (represented by B100 and P100) demonstrates lower swelling and water absorption properties (Table 2). As these groups contain the highest percentage of outer bark and given that the outer bark contains higher concentrations of hydrophobic lignin and suberin, improvements in hygric properties are both plausible and expected [31]. Larch bark, in particular, displays even lower WA and TS values. However, only the difference in WA is statistically significant, likely due to the reduced content of inner bark. Conversely, samples containing bark appear to have smaller pores, leading to higher homogeneity. This might also affect water absorption when compared to samples with a higher wood content.

Notably, B100 samples exhibit greater variability across most properties compared to other groups. Since this behavior is not observed in the P100 group, it suggests that the variability is not directly caused by the outer bark. Instead, it is hypothesized that larger bark particles present within the B material may introduce areas of lower density at their edges. The correspondingly lower properties in this region then lead to higher variability.

**Figure 4:**
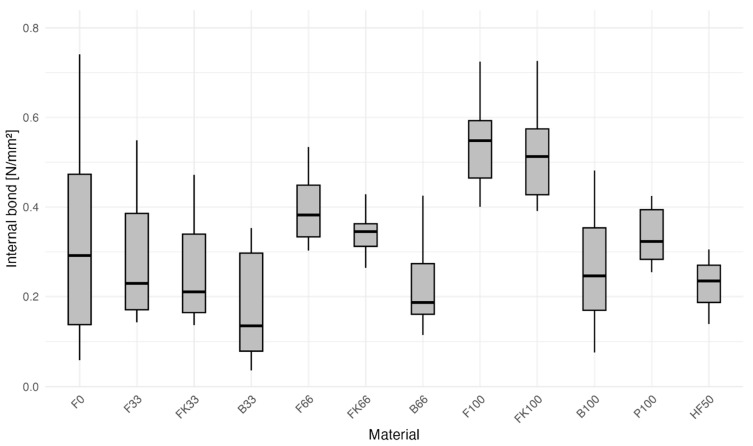
Boxplot of internal bond (IB) by group of bark-based particleboards, highlighting the symmetry of the boxes.

Increased variation is also noted in the “_33” and F0 groups concerning the internal bonding (IB) property (Figure 4). This variation is attributed to the production process, as the initial batch of boards utilized an older adhesive nearing the end of its shelf life, potentially diminishing its binding efficacy and resulting in weaker bonds in the more wood-based boards. This hypothesis is supported by the observation that the second batch, which used a fresh batch of glue, did not exhibit the same bonding issues. Furthermore, the HF50 group also demonstrates reduced IB in comparison to the reference study [15]. However, this effect was not consistent across all groups, as bark-rich groups seem not to suffer as much. Interactions with extractives in the bark or with wood particles remain an alternative explaination.

The F100 and FK100 groups demonstrate internal bonding (IB) values that surpass those of the reference Group F0, most likely due to the previously described issue with the glue. When isolating the data from the second batch alone, the F100 and F0 groups show equal performance, although it must be acknowledged that the sample size is insufficient for definitive statistical conclusions. Contrary to previous studies, which associated an increased bark content with a reduction in IB, our findings suggest a tendentially reversed trend [1,15]. It seems that the lower mechanical performance typically associated with increased bark content may be offset by optimized material preparation methods, like extractive reduction and enhancements in fibrous morphology. This strategy is commonly employed in products like medium-density fiberboard (MDF) to achieve better performance compared to particle boards. Examination of Boxplot 3 reveals a slight asymmetry in most of the boxes in relation to their median, likely attributable to batch variations, which may pose challenges for interpretation. Apart from IB itself, bark-based boards seem to be able to endure almost double the strain compared to more wood-based counterparts.

Both modulus of rupture (MoR) and modulus of elasticity (MoE) exhibit a negative correlation with increasing bark content. This trend aligns with findings by Blanchet et.al., who reported an MoR of 8.3 N/mm^2^ and an MoE of 1300 N/mm^2^ in samples with 12% urea–formaldehyde resin and a density of 0.75 g/cm^3^ using 100% hammer-milled spruce bark [1]. However, considering that most boards today are laminated and that the bending properties are significantly affected by the surface properties, the reduction in MoE and MoR may be acceptable in combination.

Thickness swelling (TS) also decreases with an increase in wood content, with the FK groups being a slight outlier. Their larger, more voluminous particle shape creates numerous pathways for water absorption, causing disproportionate swelling. The absence of glue within these fiber clumps further intensifies this issue, as glue could act as a barrier to water or slow its absorption. This leads to asymmetric swelling in certain areas of the board, although the overall swelling is not significantly different from the F group. Consistent with the literature, outer bark seems to improve water absorption and thickness swelling in comparison to materials with a higher proportion of inner bark [3].

In contrast to other studies referenced in Table 3, TS is generally significantly higher than the normative values specified for non-load-bearing particle boards (P4) [32]. Compared with the findings of Kain et al. (2020), the results of this study match or surpass them across various properties under very similar conditions [8]. Compared with Blanchet et al., it is observed that aside from IB, bark contributes to lower performance, which could be due to the 50% higher amount of glue used in board production, as glue is known to enhance board properties [1]. Compared to the HF50 group, Xing et al., who studied the integration of bark particles in MDF, reported almost double the values for every property [15]. The minor difference in density (from 0.75 g/cm^3^ to 0.85 g/cm^3^) does not fully account for this discrepancy. However, the previously mentioned issues with the older glue batch could be partly responsible for the observed differences.

### 3.2. Assessment of Material Type on the Particleboard Properties

**Figure 5:**
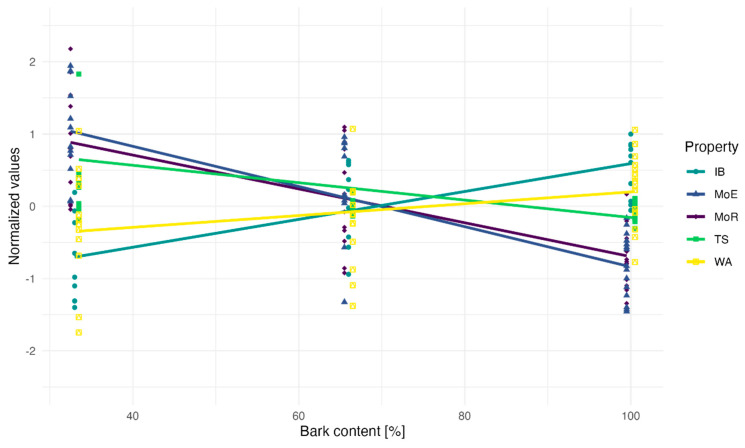
Correlation towards level by property corrected for density.

One potential confounding factor for mechanical properties is the density, which, as noted in the previous chapter, varies slightly. Therefore, density adjustments are necessary before interpreting the results. To assess the relative impact of density on the measured properties, the data presented in Figure 5 were normalized to accommodate the different scales of the variables. An impact of density is observed across all properties, with the notable exception of water absorption, where its influence is not significant after density correction (Table 4). The most substantial negative impact of density is on MoE, followed by MoR. Internal bonding (IB) displays a positive correlation with increased bark content, while in the previously referenced studies the correlation was negative. This unusual effect could be explained by the more favourable morphology of the fibers, separation of the anatomical layers, binderless recombination of smaller fibers, and removal of extractives during the processing.

In terms of thickness swelling, the effect of density remains highly significant; however, its influence on hygric properties appears to be less pronounced than on mechanical properties. This is corroborated by the water absorption data, in which the level of bark content is not a significant factor. Nonetheless, it should be considered that this might be due to variations within the material groups, as the B groups exhibit significantly lower absorption values, which could potentially skew the conclusions.

To address violations of the assumptions inherent in multiple linear regression analysis, the model types were modified to include robust linear models (RLM) and Cochrane–Orcutt corrections to compensate for autocorrelations. The R^2^ values for the corresponding properties indicate that density and bark content (level) can, on average, account for about 50% of the variance in mechanical properties, but only 12% of the variance in hygric properties.

After adjusting for both density and bark content, the F groups displayed a statistically significant difference in MoR compared to other groups. In contrast, MoE revealed that the FK groups significantly underperformed. This suggests that the larger fiber clumps in the FK groups result in reduced MoE, as reported in similar cases as well [11]. Conversely, for MoR, the more fibrous nature of the F group seems to confer an advantage. Regarding IB, the stiffer nature of the B group appears to be a drawback, likely due to the physical characteristics associated with the outer bark. However, this same property provides the B group with a benefit in terms of water absorption. In the case of thickness swelling, each group exhibited distinct performance levels.

In the previous analysis, only the variations among the different bark groups were considered. To extend the comparison to include the reference samples F0 and the link groups, a modified analytical approach was adopted. This new model excludes all groups where the bark content (level) is neither 0% nor 100%, while still correcting for density. Additionally, adjustments were made to the model to rectify violations of its assumptions.

For the reference F0 group, significant differences are observed when compared to most other groups, with the exception of IB. In IB, the groups predominantly comprising inner bark (F and FK) exhibit enhanced strength, as has been previously discussed. Moreover, the groups rich in outer bark (*p* and B) demonstrate increased resistance to water absorption, which could be attributed to a higher content of suberin in these anatomical regions, rendering them more hydrophobic.

The link group P, primarily composed of outer larch bark, does not adhere to the pattern observed in the B group. This discrepancy underscores the importance of bark’s anatomical structure and the impact of preparation methods. As the material is immutable, optimizing the preparation process (such as fractionation) presents a promising area for future research.

Cross-referencing the significance tests from Table 4 and Table 5 corroborates the findings for the properties of MoR, IB, and TS. In terms of MoE, the previously noted disparity between the B and FK groups has diminished to a non-significant level, underscoring a marginal advantage for the F group in this aspect. Regarding WA, the variance within the majority of the groups is such that the differences in WA are statistically significant across all comparisons, illustrating pronounced variability in this hygroscopic property among the groups.

### 3.3. Formaldehyde Content

The data summarized in Table 6 reveal a discernible trend: as the bark content in the sample increases, there is a significant and linear decrease in the formaldehyde content, particularly when measured at a standardized 6.5% moisture level. While this trend is less pronounced at 0% moisture content, it remains evident. This finding is of considerable significance for evaluating the impact on indoor air quality, as it corroborates prior research indicating that bark can sequester formaldehyde due to the ability of bark and its contained tannins to bind formaldehyde [4,5]. This suggests that bark may serve as an alternative to traditional methods for mitigating free formaldehyde emissions in board materials. However, the absolute levels of formaldehyde differ drastically from the studies by [4,5], who reported formaldehyde contents between 3.5 and 0.5 mg/100 g and between 5 and 1 mg/100 g for UF bonded larch bark boards, respectively. Since both the wood particles and the UF adhesive were obtained from the company Kaindl, which exclusively produces boards that conform with the Super E0 standard (perforator values below 1.5 mg/100 g) [4], there is likely an issue with either the production or the measurement of the samples. The fiber production could also have contributed, as the removed extractives are known to interact with formaldehyde. Since formaldehyde is not the main focus of this study, the exact reasons remain to be explored in future research. The relative conclusions, however, are still valid.

Furthermore, the data indicate that samples with higher bark content exhibit a greater equilibrium moisture content compared to those primarily composed of wood, or that the bark-containing samples may not acclimate to ambient conditions as rapidly as wood. A 3% higher equilibrium moisture content of bark panels was also reported by [8,11]. This observation has important implications for interpreting mechanical property results. Since the strength attributes, like MoR and MoE, of biogenic materials are contingent on the materials’ moisture content, elevated moisture levels typically undermine material strength. It is important to note that the sample preparation for formaldehyde testing differed from that for mechanical strength testing, and it is not anticipated that the moisture variation in boards during mechanical testing was as substantial. Even so, this suggests the need to include moisture as a covariate when comparing wood and bark in future research.

## 4. Conclusions

In summary, this investigation of the attributes of bark-based composite materials revealed several key findings:

A key discovery is that the bark content in certain tested materials exhibits a neutral or even beneficial effect on IB strength, challenging the prevailing consensus in the existing literature. This enhancement in IB strength may result from an increase in material homogeneity, the prevalence of self-bonding within clumps, removal of extractives during the fiber production, and refinement of morphological characteristics.

Consistent with established studies, the other mechanical properties of the composites examined adhere to the expected trends associated with bark-based materials. Nevertheless, this study brings to light the distinct attributes of inner and outer bark. It appears that the fibrous nature of inner bark is more conducive to strength-related properties, while the outer bark excels in hygric properties, possibly due to its inherent protective function against environmental factors such as moisture.

The presence of bark fibers is shown to reduce the formaldehyde content within the boards, positioning them as a viable substitute for traditional formaldehyde-capturing substances. Concurrently, this research underscores the differences in moisture content between bark-based and wood-based particle boards, accentuating the importance of considering moisture as a covariate in future studies due to its significant effect on mechanical properties.

These insights collectively highlight the potential of bark, specifically from select layers of the tree, as an efficient and cost-effective material. Processed adeptly, bark can offer improved internal bonding and lower formaldehyde emissions, notwithstanding some compromises in mechanical robustness. Future research should aim to refine the processing and preparation techniques for bark-based materials to mitigate these compromises and fully leverage the innate potential of bark in the production of wood composite materials.

## Figures and Tables

**Table 1:** Design of experiment showcasing the number of panels with bark content levels and bark materials: bark fibers (F), bark fiber clumps (FK) and outer bark (B).

Bark Content [%]	Bark Material		
	F	FK	B
100	3	3	3
66	3	3	3
33	3	3	3
0	3	0	0

**Table 2:** Results (**mean ± coefficient of variation [%]**) by group and properties. MoR = Modulus of Rupture [N/mm^2^], MoE = Modulus of Elasticity [N/mm^2^], IB = Internal Bond [N/mm^2^], TS = Thickness Swelling after 24 h (%) and WA = Water absorption after 24 h (%) *n* = 28, 3, 3, 4, 16, 16.

Group	MoR	MoE	IB	TS	WA
F0	10.12 ± 22%	2095 ± 16%	0.33 ± 68%	0.54 ± 74%	0.84 ± 8%
F33	8.08 ± 17%	1615 ± 16%	0.29 ± 53%	0.48 ± 83%	0.81 ± 12%
FK33	7.81 ± 18%	1615 ± 15%	0.25 ± 44%	0.33 ± 11%	0.75 ± 6%
B33	6.99 ± 20%	1614 ± 17%	0.18 ± 67%	0.35 ± 16%	0.75 ± 13%
F66	6.61 ± 27%	1122 ± 35%	0.39 ± 19%	0.31 ± 11%	0.8 ± 14%
FK66	6.01 ± 24%	965 ± 34%	0.35 ±1 6%	0.36 ± 8%	0.83 ± 7%
B66	5.46 ± 22%	1127 ± 28%	0.23 ± 43%	0.29 ± 12%	0.64 ± 8%
F100	6.28 ± 19%	865 ± 23%	0.55 ± 21%	0.27 ± 9%	0.76 ± 8%
FK100	5.06 ± 20%	680 ± 30%	0.53 ± 21%	0.33 ± 12%	0.85 ± 11%
B100	4.35 ± 38%	715 ± 47%	0.27 ± 45%	0.21 ± 21%	0.47 ± 28%
P100	4.44 ± 23%	767 ± 32%	0.34 ± 18%	0.16 ± 17%	0.37 ± 17%
HF50	10.4 ± 4%	1529 ± 4%	0.23 ± 36%	0.31 ± 5%	0.76 ± 6%

**Table 3:** Comparison with other studies. (−) indicates a linear negative correlation with increasing bark content; (+) indicates a linear positive correlation with increasing bark content.

Author	Resination Factor [%]	Bark Amount [%]	Density [g/cm^3^]	MoR [N/mm^2^]	MoE [N/mm^2^]	IB [N/mm^2^]	TS [%]	Material
This study	8%	100	0.75	6.28 (−)	823 (−)	0.56 (+)	27% (−)	spruce bark (F100)
[1]	12%	100	0.75	8.3 (−)	1300 (−)	0.372 (−)	14% (+ −)	hammermilled spruce bark
This study	8%	50	0.75	10.4 (−)	1529 (−)	0.23 (+ −)	0.31 (−)	HF50
[15]	8%	50	0.85	28 (− −)	2800 (−)	0.52 (−)	19.3% (−)	MDF with bark in core layer
P100	8%	100	0.69	4.4 (−)	767 (−)	0.34 (−)	0.16 (−)	P100, larch
[8]	8%	100	0.55	2.85	528	0.33	0.29	shredded bark (spruce and larch)
P2 (according to [32])	-	-		11	1600	0.35	15% *	

* Values for a particleboard type P4 [32].

**Table 4:** Impact of density and level on properties and difference between groups after correcting for the influence of density. Ols = Ordinary least square regression, rml = robust linear regression.

			Significance		*p*-Value		
Property	Model	R^2^	density	level	F-B	FK-B	FK-F
MoR	ols + cochrane_orcutt	0.364	0.000	0.000	0.000	0.950	0.000
MoE	ols + cochrane_orcutt	0.548	0.000	0.000	0.656	0.002	0.000
IB	rml + cochrane_orcutt	0.546	0.000	0.000	0.000	0.000	0.346
TS	rlm	0.101	0.133	0.001	0.076	0.289	0.869
WA	rlm	0.139	0.001	0.459	0.000	0.000	0.265

**Table 5:** Difference between residuals or boards with only one material type corrected by density.

Property	*p*-Value									
	F0-F	F0-FK	F0-B	F0-P	P-F	P-FK	P-B	F-B	F-FK	FK-B
MoR	0.000	0.000	0.000	0.000	0.762	0.282	0.120	0.002	0.013	0.998
MoE	0.000	0.000	0.000	0.000	0.026	0.000	0.004	0.956	0.016	0.082
IB	0.000	0.009	1.000	0.734	0.009	0.132	0.680	0.000	0.887	0.003
TS	0.003	0.039	0.000	0.000	0.495	0.180	0.962	0.844	0.935	0.427
WA	0.371	0.866	0.000	0.000	0.000	0.000	0.003	0.000	0.023	0.000

**Table 6:** Average formaldehyde content of boards of F groups with exicator method. Triple evaluation, 110 g each.

Sample	Moisture Content [%]	Formaldehyde Content at 0% m.c. [mg/100 g]	Formaldehyde Content at 6.5% m.c. [mg/100 g]
F0	5.07	20.92	24.81
F33	6.16	19.84	20.65
F66	8.00	19.74	15.71
F100	9.64	18.24	10.54

## Data Availability

The original contributions presented in the study are included in the article, further inquiries can be directed to the corresponding author.
